# The effect of gender status on the growth performance, carcass and meat quality traits of young crossbred Holstein-Friesian×Limousin cattle

**DOI:** 10.5713/ajas.20.0085

**Published:** 2020-07-02

**Authors:** Paulina Pogorzelska-Przybyłek, Zenon Nogalski, Monika Sobczuk-Szul, Martyna Momot

**Affiliations:** 1 Department of Cattle Breeding and Milk Evaluation, Faculty of Animal Bioengineering, University of Warmia and Mazury in Olsztyn, ul. Oczapowskiego 5/137, 10-719 Olsztyn, Poland

**Keywords:** Beef Cattle, Bulls, Steers, Heifers, Meat Quality, Sensory Evaluation

## Abstract

**Objective:**

The objective of this study was to compare growth performance, carcass traits and meat quality in young bulls, steers and heifers produced by crossing Limousin bulls with Holstein-Friesian cows, fattened semi-intensively and slaughtered at 18 months of age.

**Methods:**

Thirty-one young calves were reared in a conventional production system, and were fed milk replacer, hay and concentrate. At 6 months of age, the animals were divided into groups based on gender, and were fed a total mixed ration composed of grass silage, concentrates I and II in a semi-intensive production system. At the end of the fattening period (18 months), the animals were slaughtered, carcass quality was evaluated, and samples of *musculus longissimus thoracis* were collected to determine the proximate composition and quality of meat.

**Results:**

Bulls were characterized by the highest percentage share of the most valuable cuts in the carcass, and three-rib sections from bull carcasses had the highest lean meat content with low intramuscular fat content (0.93%). No significant differences in carcass conformation, dressing percentage or the percentage share of round in the right half-carcass were found between bulls vs. steers and heifers. Heifers and steers had higher carcass fat content than bulls, which had a positive influence on the sensory properties of beef. In comparison with the meat of bulls, the meat of steers and heifers was characterized by more desirable physical properties and sensory attributes (water-holding capacity, shear force, color lightness, aroma, juiciness, tenderness, flavor).

**Conclusion:**

Under the semi-intensive production system, heifers and steers had higher carcass fat content than bulls, which had a positive effect on the sensory properties of beef. Bulls are better suited for intensive systems, which contribute to improving the quality of their meat. The results of this study may encourage producers to breed steers and heifers for beef.

## INTRODUCTION

The growth performance and carcass attributes of cattle are influenced by gender (male, female, castrated) [1,2]. Females are more affected than males due to their higher precociousness, whereas steers maintain an intermediate position. Differences in carcass conformation and fat content might also affect other meat quality parameters such as pH, color and tenderness. In Europe, beef is produced from cows, heifers, steers and young bulls of beef and dairy breeds. The percentage of their contribution varies across countries. For instance, more than 50% of beef comes from cows in France and from young bulls in Poland [3,4]. As a result, consumers have no access to beef from steers and therefore have no opportunity to appreciate its high quality. Due to the low profitability of beef cattle production, a continuous increase in the prices of protein supplements (which is a long-term trend) and high, fluctuating grain prices, the animals are fed mostly cheap farm-made feeds based on grass silage supplemented with concentrates. Grazing is often associated with low-energy diets and low energy intake, furthermore producing bulls characterized by low fatness, muscles with poorer color stability and more intense flavor, and tougher meat. Semi-intensive production systems are dedicated to females and steers rather than bulls. From a cattle breeder’s perspective, the advantage of steers is that they do not require very high energy concentrations in the ration, and are less sensitive to restriction or changes in dietary protein levels than bulls. In comparison with bulls, steers are also more docile and easier to handle because castration lowers their blood testosterone levels. Thus, they can be grazed together with other animals, including heifers. In Poland, heifers account for approximately 15% of all cattle slaughtered, and 312,200 heifers were slaughtered in 2018 [5].

The aim of this study was to compare growth performance, carcass traits and meat quality in young bulls, steers and heifers produced by crossing Limousin (LM) bulls with Holstein-Friesian (HF) cows, and slaughtered at the same age.

## MATERIALS AND METHODS

### Animals

The experiment was performed at the Agricultural Experiment Station in Bałcyny (53°35′29″ N; 19°50′58″ E; Poland), on 31 animals, including 10 young crossbred bulls, 10 steers and 11 heifers, produced by crossing HF cows with LM bulls. Calves of known origin, which were purchased at 2 or 3 weeks of age, underwent a 7-day quarantine period. Bloodless castration of 10 bulls was carried out using a rubber elastrator. The calves were kept in a group pen. They were fed milk replacer from automatic feeders, hay and concentrate, followed by grass silage. At 6 months of age, the animals were divided into groups based on gender, and were placed in free-stall group pens in a fattening facility. After a 30-day adaptation period, the animals were fed a total mixed ration (TMR) composed of grass silage, concentrate I (to body weight of 300 kg) and concentrate II (at body weight above 300 kg) in a semi-intensive production system (Table 1). The proportion of silage to concentrate in the ration was 75% to 25%. The concentrate consisted of ground triticale, rapeseed meal and minerals-vitamin premix. A commercial mineral-vitamin premix for fattening cattle (product code 7619; Cargill Poland Ltd., Warsaw, Poland) was composed of the following ingredients (per kg of premix): Ca, 235 g; Na, 79 g; P, 48 g; Mg, 28 g; Fe, 500 g; Mn, 2,000 mg; Cu, 375 mg; Zn, 3,750 mg; J, 50 mg; Co, 12.5 mg; Se, 12.50 mg; vitamin A, 250,000 IU; vitamin D_3_, 50,000 IU; vitamin E, 1,000 mg; dl-alpha-tocopherol, 909.10 mg. TMR, administered from a self-propelled feed cart (Seko, Curtarolo, Italy), was delivered to feeding stations twice daily (at 8:00 am and 4:00 pm). The animals were fattened until 18 months of age. The study was conducted in 2013 through 2014 upon the approval of the Local Ethics Committee for Animal Experimentation (decision No. 121/2010).

### Slaughter and carcass traits

The animals were weighed to the nearest 0.5 kg at the beginning (180 days) and at the end of the fattening period. Average daily gains were calculated. Upon reaching the slaughter age, the animals were transported over a distance of 90 km to the abattoir where they were kept in lairage for 15 to 20 hours prior to slaughter, in individual boxes with free access to water. The animals were stunned, slaughtered, dressed, and halved along the spine into two half-carcasses that were then chilled for 96 hours at 4°C. Electrical stimulation was not applied to the carcasses. Half-carcasses were weighed to the nearest 0.5 kg on an automated on-line scale (hot carcass weight [HCW]), and conformation and fat cover were evaluated based on the EUROP system criteria by a trained grader. The protocol for animal research was approved by the Ethics Committee of the University of Warmia and Mazury in Olsztyn. All slaughter and post-slaughter processes were carried out in accordance with the current meat industry regulations [6].

Carcass dressing percentage (percentage ratio of carcass weight to live body weight at slaughter) was calculated. Ninety-six hours post mortem, three-rib (10th to 12th rib) sections were sampled from the right half-carcasses (two cuts through a half-carcass, perpendicular to the spine, between the 9th and 10th, and the 12th and 13th thoracic vertebrae).

The surface area of *m. longissimus thoracis* (LT) was outlined on wax paper, between the 9th and 10th thoracic vertebrae, and was measured with a planimeter.

Half-carcasses were divided into primal cuts and five most valuable cuts, passing through anatomical points of the half-carcass, i.e. shoulder (the upper portion of the front leg without the shoulder cartilage), fore ribs (separated by an anterior cut along the neck cutting line between the last cervical vertebra and the first thoracic vertebra; a posterior cut along the line between the 6th and 7th thoracic vertebrae; an inferior cut along the cutting line separating the thin flank, from the head of the first rib to the bottom edge of the iliocostalis), best ribs (separated by an anterior cut along the line between the 6th and 7th thoracic vertebrae; a posterior cut along the line between the last and last but one thoracic vertebrae; an inferior cut along the cutting line separating the thin flank, as above), loin (separated by an anterior cut along the line between the last and last but one thoracic vertebrae; a posterior cut along the line between the last lumbar vertebra and the first sacral vertebra; an inferior cut along a straight line, 5 to 7 cm from the muscles in the back) and round of beef (separated by an anterior cut along the line between the last lumbar vertebra and the first sacral vertebra, along the perimysium of the quadriceps femoris; an inferior cut along the cutting line separating the shank at the stifle joint), were weighed and their percentage shares of the right half-carcass were estimated.

Three-rib cuts were dissected, and the percentage content of soft tissues (lean meat, fat, tendons) and bones was determined.

### Chemical composition, physical and sensory properties of *m. longissimus thoracis*

During carcass dressing, LT samples were collected from the right half-carcasses to evaluate beef quality. Meat samples weighing approximately 300 g were packaged in polyamide/polyethylene (PE) vacuum bags at an ambient temperature of around 4°C, under standard industrial conditions. Meat color was evaluated immediately in the laboratory, based on the values of CIELAB coordinates, L*, a*, and b* [7]. Color space parameters L*, a*, and b* were measured three times by the reflectance method, using a MiniScan XE Plus spectrocolorimeter (HunterLab, Reston, VA, USA) with standard illuminant D65, a 10° standard observer angle and a 2.54-cm-diameter aperture. The devise before measurements was calibrated using white and black tiles. The measurements were performed at different points over the muscle cross-section area. Color measurements were performed on meat samples stored for 30 min at 4°C, covered with foil permeable to O_2_ and impermeable to H_2_O. After color measurements, each meat sample was divided into two portions: the first portion was used to determine the proximate chemical composition and physicochemical properties of meat, and the other portion was used to evaluate the sensory attributes of meat.

The analysis of the proximate chemical composition of meat included the determination of dry matter, total protein, crude fat and ash, according to the procedure proposed by Wajda et al [8]. The following physicochemical properties of meat were determined: ultimate pH, water-holding capacity and Warner-Bratzler shear force (WBSF). The value of pH48 was measured after 48 h of carcass chilling, in LT, between the 10th and 11th thoracic vertebrae. Water-holding capacity was determined based on natural drip loss and cooking loss. To estimate natural drip loss, approx. Meat samples (20 g) were packaged in PE string bags and placed in an incubator at a temperature of 4°C±1°C. After 24 h, the samples were dried and weighed again within an accuracy of 0.001 g. Natural drip loss was calculated as the difference between sample weights before and after cold storage. Cooking loss was determined according to the method proposed by Honikel [9]. Meat samples were weighed, they were packaged in plastic bags and placed in a water bath at a temperature of 80°C for 1 h. Then the samples were cooled for 30 min under running water, dried and weighed again to determine their weight after cooking. Cooking loss was calculated as the difference between sample weights before and after heat treatment. WBSF values (N) were measured using an Instron 5542 universal testing machine (Instron, Norwood, MA, USA) equipped with a shear blade. Cylindrical core samples (1.27 cm in diameter, approx. 40 mm long) were cut out with a cork borer in the direction of muscle fibers. The shear blade (V-shaped, with a triangular aperture of 60°) was applied perpendicularly to the fiber direction at a crosshead speed of 200 mm/min. The test was performed at room temperature (approx. 18°C). The data were analyzed using Bluehill 3 software (Instron, USA).

To evaluate the sensory properties of beef, 200 g samples were cut out across the muscle fibers, and were cooked in a 0.6% NaCl solution (meat to solution weight ratio of 1:2) at a temperature of 96°C (±2°C). Pasteurization was carried out until the temperature inside the sample reached 75°C. The samples were evaluated based on Polish Standard [10]. The sensory attributes of coded meat samples (aroma, flavor, juiciness, tenderness) were evaluated on a five-point scale (where 1 and 5 denoted the minimum and maximum score, respectively) by five trained panelists selected based on their flavor sensitivity. The samples were presented to the panelists at room temperature (approx. 20°C), in fluorescent light. Each panelist received coded samples in the random order, and each sample was tested by all panelists.

### Statistical analysis

Three cattle groups were analyzed: bulls (n = 10), steers (n = 10), and heifers (n = 11). The data were processed statistically by analysis of variance (ANOVA) and Tukey’s honest significant difference test at a significance level of 5%. All calculations were performed with the use of Statistica 10 software (StatSoft Inc., Tulsa, OK, USA)

## RESULTS AND DISCUSSION

Semi-intensive feeding was reflected in the fattening performance of bulls, steers and heifers (Table 2). After 11 months of fattening, bulls were characterized by the highest average body weight, whereas the lowest average body weight was noted in heifers (p<0.05). There were no significant differences in dry matter intake per kilogram of animal body weight. The differences in body weight at the end of fattening were reflected in HCW. Steers had HCW of 260.0 kg, which was an intermediate value between those determined in bulls and heifers. The less intensive grass-based system had no negative effect on the production potential of bulls whose average daily gain was highest (0.846 kg). A higher growth rate of males, compared with females, including castrated males vs females, has been well documented in previous studies. According to de Araujo Marques et al [11], the low final body weight of heifers is due to lower muscle deposition in this gender. Despite semi-intensive fattening, fat cover score was highly significantly higher in heifers (7.7 points) than in bulls (4.8 points) and steers (5.0 points). This is consistent with the findings of Bureš and Bartoň [1] who demonstrated that crossbred heifers of late-maturing breeds slaughtered at 18 months of age had considerably higher carcass fat content than their counterparts slaughtered at 14 months of age. Considerable carcass fatness in heifers suggests that crosses of the late-maturing HF breed and the medium-maturing LM breed should be slaughtered at an earlier age. In the present study, higher fat scores were expected in steers. In a previous experiment conducted by Nogalski et al [12], steer carcasses had significantly higher fat content than bull carcasses. An interaction between gender and body weight before slaughter was noted for carcass fatness because the increase in fat content with increasing body weight was considerably higher in steers than in bulls. In the current study, no significant differences in fat cover scores were found between bulls and steers (Table 2).

The slaughter value of cattle and carcass quality are related to the percentage share of cuts with high market value in the carcass. According to Choroszy et al [13], beef carcasses classified into higher conformation classes in the EUROP grading system have higher weight of five primal cuts. In the present study, no significant differences in carcass conformation were noted between genders. However, bulls had a higher percentage share of the most valuable cuts in the carcass than steers (p<0.01) and heifers (p<0.05), and a higher percentage share of LT in the right half-carcass than steers (p<0.05). Bulls have high carcass value due to their capacity to deposit muscle [14]. Testosterone, the principal androgen produced by interstitial cells in the testicles (particularly in sexually mature males), stimulates muscle development and promotes nitrogen retention [15]. In bulls castrated before reaching sexual maturity, androgen production is inhibited, growth rate slows down, and fat deposition increases [16]. In this study, no significant differences in dressing percentage, the percentage share of round in the right half-carcass or LT area were found between genders. Heifers were characterized by the highest dressing percentage, but the noted differences were not statistically significant. The value of 57.54%, determined in our study, is higher than that reported by Węglarz [17] who found that dressing percentage was significantly higher in heifers than in bulls (53.3% vs 52.5%). Similar values of dressing percentage in heifers and steers vs. bulls can be attributed to their high carcass conformation scores (7.4 points in heifers and 7.2 points in steers vs 7.3 points in bulls) and greater predisposition to deposit fat (higher fat cover score).

Tissue composition is another determinant of beef carcass quality (Table 3). Significant differences in the percentage content of lean meat, fat and bones in three-rib cuts were found between bulls, steers and heifers. In comparison with heifers, three-rib cuts from bull carcasses and steer carcasses had significantly higher lean meat content, and the difference between bulls and steers was small (2.68%). The difference in the fat content of three-rib cuts between bulls and steers is consistent with the difference in their fat cover scores in the EUROP grading system. In heifers, an increase in carcass fat content was accompanied by a decrease in carcass lean content. Carcass fat content was 5.15% higher in steers than in bulls, but this value is not indicative of excessive fatness; on the contrary, it could have a beneficial influence on the sensory attributes of beef (Table 5).

An analysis of the proximate composition of LT revealed that gender had no effect on the ash and total protein content of LT, although such correlations were observed by Rotta et al [14]. Ash is important for the supply of sodium, potassium, phosphorus and magnesium which are of great nutritional importance to humans. In the current experiment, females had fatter carcasses (Table 2) and higher intramuscular fat (IMF) percentage (Table 4) than males. According to Priolo et al [18], higher carcass fatness is associated with a slower cooling rate of muscles resulting in a faster pH decline. Slow cooling combined with a low pH of muscles enhances protein denaturation, leading to an increase in color lightness L* [19]. There is a linear relationship between the active acidity of meat and L* values: an increase in the concentration of hydrogen ions (pH) in meat contributes to a decrease in color lightness, whereas L* values increase with decreasing pH levels [20]. Meat from heifers had higher values of color parameters L*, a*, b*, compared with meat from bulls and steers. Intramuscular fat is the chemically estimated fat content of meat, and the terms marbling and IMF are often used interchangeably [21]. Intramuscular fat is lighter in color than lean beef, and therefore its presence in muscles could contribute to an increase in L* values [2]. Meat color plays an important role in a consumer’s purchase decisions (Table 4).

The ability of muscles to hold water (water-holding capacity) is primarily determined by their pH. As the pH of beef increases, its water-holding capacity and the rate of heat transfer increase as well [22]. In the current study, meat from heifers had higher water-holding capacity than meat from bulls, and an intermediate value was determined in steers. The amount of water captured and retained in meat before and after cooking influences its juiciness and, consequently, palatability as well as the saleable weight of the product [23]. Greater cooking loss of muscles from bulls vs. steers and heifers was noted by Zhang et al [24]. In studies by Węglarz [17], and Hanzelkova et al [25], beef from bulls was characterized by greater cooking loss than beef from heifers. Higher fat deposition in heifers improved the water-holding capacity of meat whereas greater fat loss during cooking increased cooking loss [26]. In the present study, gender had no influence on cooking loss.

Studies investigating trends in beef production should focus not only on improving slaughter value and carcass traits, but also the quality of beef so as to meet rising consumer expectations. Consumer research conducted until the 1990s revealed that tenderness was the most important attribute driving consumer liking. However, more recent studies have shown that as overall tenderness improved and tenderness variation decreased, flavor has become a more important driver of beef consumer liking. In general, as long as tenderness and juiciness are at acceptable levels, flavor is the main driver of overall liking [27,28]. In the present study, beef from steers and heifers scored higher for flavor (4.86 in both cases) than beef from bulls. More significant differences between animals of different sex categories were noted for tenderness. The Warner-Bratzler shear test is the most widely used instrumental test for meat tenderness evaluation. According to research conducted several decades ago, gender has no significant effect on WBSF values [29]. Later studies [25,30] revealed that gender is a significant determinant of beef tenderness regardless of cattle breed or aging time, and that meat from bulls is generally tougher than meat from heifers. This may be explained by gender differences between animals of similar age and physiological maturity. As muscle tissue matures, the structure of collagen becomes more compact and its solubility decreases. This is an important consideration because the solubility of intramuscular collagen is positively correlated with meat tenderness and sensory properties [31]. The lower tenderness of beef from bulls can be attributed to its higher collagen content, compared with beef from steers and heifers [24,32]. Due to its higher tenderness, meat from females is more appreciated by consumers than meat from males. On the other hand, castration improves beef quality because it increases carcass fat content. Castrating bulls generally increases IMF content in several breeds, including Korean cattle and Holsteins, and differences in adiposity are reflected in the eating quality of beef. Consumers from European countries, including Germany and France, prefer lean beef whereas in Korea, Japan, the USA and Australia, IMF is more important to beef quality grade due to considerable differences in IMF content between cattle breeds [33]. In the present study, samples of meat from heifers had lower WBSF values compared with samples collected from bull and steer carcasses. This is consistent with the results cited above, but other studies revealed no significant differences in WBSF values or tenderness between heifers and steers [34]. Beef from heifers was characterized by significantly higher tenderness in comparison with meat from bulls and steers (Table 4). Higher juiciness and flavor scores for beef from heifers and steers, compared with beef from bulls, can be attributed to the higher IMF content of muscles in heifers and steers than in bulls, in which IMF content was only 0.93% (Table 5). Intramuscular fat is believed to positively influence meat tenderness and juiciness. In our experiment, steer carcasses had optimal IMF content (2.21%), which had a beneficial effect on the aroma and flavor of beef. Such an IMF content of beef meets the preferences of modern consumers who look for low-fat products. In the current study, heifers received the highest scores and bulls received the lowest scores for the sensory attributes of beef, and intermediate values were noted in steers. These observations agree with the findings of Bureš and Bartoň [1] who reported higher sensory scores in heifers vs bulls and steers. However, in another study [34] beef from steers received higher sensory scores than beef from bulls and heifers.

Recent research has demonstrated that tenderness evaluation by untrained consumers is a better procedure for assessing the sensory properties of beef, compared with evaluation by trained consumers or instrumental measures [35]. The initial MSA model was developed in Australia using untrained consumer scores and production and processing data from beef samples. The system is updated on a regular basis to reflect current consumer preferences and production methods, which is one of its advantages over instrumental methods and evaluation by trained consumers. For instance, the weights of the four sensory scores (tenderness, juiciness, flavor liking, overall liking) used to determine eating quality (MQ4) were modified to apply equal weightings to flavor liking and tenderness, in order to reflect changing consumer preferences. Such an approach allows beef producers to maintain flexibility in responding to changing consumer tastes and making the relevant management decisions. In the present study, the above MSA grading scheme was used to determine the effect of gender on the eating quality of beef. The results were consistent with our findings. Beef from young bulls had slightly lower MQ4 scores than beef from steers and females [3].

## CONCLUSION

Under the semi-intensive production system, heifers and steers had higher carcass fat content than bulls, which had a positive influence on the sensory properties of beef. The too low IMF content of meat from bulls (0.93%), which affected its sensory properties, was associated with the diet. Bulls are better suited for intensive systems than steers and heifers. A higher proportion of concentrate in the ration could have a beneficial influence on the fattening performance and carcass characteristics of bulls. Beef from steers and heifers can meet rising consumer expectations because it is characterized by more desirable physical properties and sensory attributes (water-holding capacity, shear force, color lightness, aroma, juiciness, tenderness, and flavor) than beef from bulls. The results of this study may encourage producers to raise steers and heifers for beef.

## Figures and Tables

**Table 1:** Chemical composition and nutritional value of the ingredients of experimental diets

Items	Silage (n = 9)	Triticale (n = 3)	Rapeseed meal (n = 3)	Concentrate I (n = 7)	Concentrate II (n = 7)
Dry matter (%)	39.7±10.9	88.1±0.22	88.7±0.21	88.4±0.7	88.6±0.8
	---------------------------------------------------------------------- On dry matter basis (%) -----------------------------------------------------------------				
Organic matter	92.0±3.1	98.1±0.25	92.7±0.27	93.2±1.3	92.5±1.8
Crude protein	14.1±1.1	13.3±0.70	38.8±0.85	18.9±1.5	16.3±0.7
NDF	56.9±5.2	19.3±0.10	31.0±0.11	20.2±1.1	18.4±0.8
ADF	38.7±5.9	4.4±0.02	22.8±0.12	7.2±0.6	3.1±0.8
DOMD	74.1±5.6	93.2±2.61	84.8±0.41	-	-
UFV	0.08±0.0	0.12±0.00	0.10±0.00	0.12±0.0	0.12±0.0
PDIN	8.2±0.6	8.9±0.03	25.9±0.09	12.2±0.2	11.2±0.5
PDIE^1)^	6.9±0.2	10.9±0.08	16.3±0.10	13.0±0.5	12.1±0.5

Mean±standard deviation.

NDF, neutral detergent fiber; ADF, acid detergent fiber; DOMD, digestible organic matter in dry matter; UFV, feed unit for meat production; PDIN, protein digested in the small intestine when rumen fermentable N is limiting; PDIE, protein digested in the small intestine when rumen fermentable energy is limiting.

1) Fermentation characteristics of silage: pH 4.8±0.3; lactic acid, 5.4%±2.0% dry matter; volatile fatty acids, 2.7%±0.5% dry matter; water soluble carbohydrates, 8.2%±4.8% dry matter; N-NH_3_ % N, 10.3±6.7; true protein 51.8%±4.6% crude protein.

**Table 2:** Fattening performance and carcass characteristics of fattened bulls, steers and heifers

Items	Bulls	Steers	Heifers	SEM	Significance
Age at slaughter (d)	554.3	554.9	560.1	2.114	ns
BW at the beginning of the fattening period (kg)	191.1	176.6	174.9	3.031	ns
BW at the end of the fattening period (kg)	507.5^a^	464.0^b^	460.5^b^	7.808	*
Average daily gain (kg)	0.846^a^	0.766^ab^	0.751^b^	0.016	*
DMI/kg BWG (kg)	6.92	7.61	7.80	0.212	ns
Hot carcass weight (kg)	280.5^a^	260.0^ab^	251.7^b^	4.558	*
Dressing percentage (%)	58.18	57.05	57.54	0.356	ns
pH_48_	5.53	5.57	5.53	0.016	ns
Conformation score^1)^ (pts)	7.3	7.2	7.4	0.231	ns
Fat cover score^2)^ (pts)	4.8^a^	5.0^a^	7.7^b^	0.369	*
Five most valuable primal cuts^3)^(%)	67.29^a^	63.31^b^	64.73^b^	0.468	*
Percentage share of round in the right half-carcass (%)	34.94	32.15	33.78	0.509	ns
Percentage share of *m. longissimus thoracis* in the right half-carcass (%)	8.31^a^	7.24^b^	7.62^ab^	0.172	*
Area of *m. longissimus thoracis* (cm^2^)	84.43	82.63	81.91	2.444	ns

SEM, standard error of the mean; BW, body weight; ns, no significant; DMI, dry matter intake; BWG, body weight gain.

1) EUROP conformation: 1 to 15, where 1 = muscling very weak; 15 = muscling outstanding.

2) EUROP degree of fat cover: 1 to 15, where 1 = very lean; 15 = very fat.

3) Five most valuable primal cuts included the shoulder, fore ribs, best ribs, loin, round.

a,b Figures in the same rows differ with different superscripts; p<0.05.

**Table 3:** Physical composition of three-rib sections

Items	Bulls	Steers	Heifers	SEM	Significance
Muscles (%)	56.46^a^	53.78^a^	48.02^b^	0.819	*
Fat (%)	16.71^a^	21.86^b^	29.35^c^	1.177	*
Bones (%)	21.88^a^	19.67^a^	16.91^b^	0.563	*
Tendons (%)	4.95	4.69	5.71	0.267	ns

SEM, standard error of the mean; ns, no significant.

a–c Figures in the same rows differ with different superscripts; p<0.05.

**Table 4:** Chemical composition and physical properties of *m. longissimus thoracis*

Items	Bulls	Steers	Heifers	SEM	Significance
Dry matter (%)	24.29^a^	26.10^b^	26.79^b^	0.270	*
Fat (%)	0.93^a^	2.21^a^	3.07^b^	0.233	*
Ash (%)	1.09	1.08	1.08	0.006	ns
Total protein (%)	21.81	22.20	21.86	0.115	ns
pH	5.61	5.56	5.51	0.020	ns
L*	34.99^a^	35.41^a^	38.71^b^	0.539	*
a*	16.93^a^	18.66^a^	24.77^b^	0.803	*
b*	12.75^a^	14.25^a^	18.67^b^	0.606	*
Cooking loss (%)	34.31	34.57	35.44	0.487	ns
Water-holding capacity (%)	1.41^a^	3.16^ab^	4.29^b^	0.376	*
WBSF (N)	49.61^a^	49.93^a^	37.91^b^	2.694	*

SEM, standard error of the mean; ns, no significant; L*, lightness; a*, redness; b*, yellowness; WBSF, Warner–Bratzler shear force.

a,b Figures in the same rows differ with different superscripts; p<0.05.

**Table 5:** Sensory quality of *m. longissimus thoracis*

Items	Bulls	Steers	Heifers	SEM	Significance
Aroma	4.50	4.68	4.50	0.084	ns
Tenderness	3.14^a^	3.29^a^	4.00^b^	0.106	*
Juiciness	3.79^a^	4.00^b^	4.00^b^	0.071	*
Flavor	4.50^a^	4.86^b^	4.86^b^	0.082	*

SEM, standard error of the mean; ns, no significant.

a,b Figures in the same rows differ with different superscripts; p<0.05.
